# High C-reactive protein-to-albumin ratio levels are associated with osteoporosis in patients with primary biliary cholangitis

**DOI:** 10.3389/fendo.2024.1415488

**Published:** 2024-05-30

**Authors:** Yanyan Li, Bo Liu, Xin Li

**Affiliations:** ^1^ Center of Integrative Medicine, Beijing Ditan Hospital, Capital Medical University, Beijing, China; ^2^ National Center for Infectious Diseases, Beijing Ditan Hospital, Capital Medical University, Beijing, China; ^3^ Department of Orthopaedics, Beijing Ditan Hospital, Capital Medical University, Beijing, China

**Keywords:** C-reactive protein-to-albumin ratio, osteoporosis, primary biliary cholangitis, association, predictive value

## Abstract

**Objective:**

Inflammation contributes to the development of metabolic bone diseases. The C-reactive protein-to-albumin ratio (CAR) is an inflammation-based marker with a prognostic value for several metabolic diseases. This study investigated the relationship between the CAR and osteoporosis (OP) in patients with primary biliary cholangitis (PBC).

**Methods:**

Patients with PBC treated at Beijing Ditan Hospital between January 2018 and June 2023 were enrolled. Logistic regression analysis was performed to investigate the factors influencing OP. The predictive value of CAR for OP was evaluated using receiver operating characteristic (ROC) curves. Moreover, a restricted cubic spline (RCS) fitted with a logistic regression model was used to analyze the relationship between CAR and OP.

**Results:**

The prevalence of OP among the patients with PBC was 26.9% (n = 82). CAR levels were higher in the OP group than in the non-OP group (0.33 (0.09, 0.61) vs. 0.08 (0.04, 0.18), P < 0.001). Logistic regression analysis showed that CAR was an independent predictor of OP in patients with PBC (odds ratio = 2.642, 95% confidence interval = 1.537-4.540, P < 0.001). CAR exhibited a good predictive ability for OP, with an areas under the curve (AUC) of 0.741. We found that individuals with CAR values > 0.1 have higher odds of OP. In addition, high CAR levels were associated with an increased prevalence of fragility fractures and high 10-year fracture risk.

**Conclusion:**

High CAR levels were associated with greater odds of developing OP, and the CAR could serve as an independent predictor of OP in patients with PBC.

## Introduction

Primary biliary cholangitis (PBC) is a chronic and progressive autoimmune disease characterized by the destruction of small intrahepatic bile ducts, leading to periportal inflammation, fibrosis, and potentially cirrhosis (1, 2). Because of the insidious nature of PBC, approximately one-third of the population may progress to cirrhosis by the time of diagnosis (3). The exact pathogenesis of PBC is complex and is not fully understood. It is thought to be associated with genetic predispositions, environmental triggers, immunological disorders, and metabolic abnormalities (4). Both the late detection of PBC and interaction of susceptibility factors increase the risk of extrahepatic complications. Osteoporosis (OP) is an extrahepatic complication in patients with PBC that increases the risk of fragility fractures and disability, negatively impacting the quality of life and survival of the population (5, 6). Compared with the age-matched general population, patients with PBC have a 4-fold and 2-fold increased risk of OP and fractures, respectively (7). Unfortunately, owing to the insidious and asymptomatic nature of bone loss, the condition often remain unrecognized until fractures occur (8). In addition, there are no specific or effective therapeutic strategies for OP (9). Therefore, early identification and timely intervention of OP are essential to prevent the progression of bone disease in patients with PBC.

However, the exact pathogenic mechanisms underlying OP remain unclear. Older age, a low body mass index (BMI), and physical inactivity are widely recognized as traditional risk factors for OP (10). In addition, several studies have reported that subclinical inflammation and poor nutritional status are involved in the development of OP and fractures (11). Many inflammatory markers such as C-reactive protein (CRP) and tumor necrosis factor α, could stimulate osteoclast activity, leading to increased bone resorption and bone loss (12, 13). Albumin is an indicator of nutritional status and plays an important role in maintaining bone health (14). A study from the United States showed that hypoalbuminemia was strongly and independently associated with OP in the general population (15).

Chronic systemic inflammation is an evident characteristic of PBC (16). Elevated levels of CRP are commonly observed in patients with PBC, particularly in those with advanced disease (17–19). Importantly, patients with chronic liver disease also exhibit impaired albumin synthesis (20). In addition, the intensity of the inflammatory response is positively correlated with the degree of hypoalbuminemia (21). Because OP and PBC share inflammation as the common risk factor, and both CRP and albumin play an important role in the inflammatory process, it is necessary to investigate the relationship between these two indicators and OP in patients with PBC. The CRP-to-albumin ratio (CAR) is a composite measure and is considered a more useful indicator than either CRP or albumin alone (22). As an inflammation-based marker, CAR has shown prognostic value in several diseases such as cardiovascular disease, autoimmune disease, and malignancy (23–25). However, the relationship between the CAR and OP development in patients with PBC remains unclear.

Therefore, this study aimed to investigate whether the CAR could be used as an effective indicator for the early assessment of OP risk in patients with PBC. These results may help clinicians identify patients at a high risk of OP early and optimize clinical judgment and management.

## Methods

### Patient population

This single-center cohort study was conducted at Beijing Ditan Hospital. Patients diagnosed with PBC between January 2018 and June 2023 were included in this study. Diagnosis of PBC was based on the criteria recommended by the European Association for the Study of the Liver (26). Exclusion criteria were as follows: (1) end-stage liver disease; (2) positive serum hepatitis virus markers or human immunodeficiency virus infection; (3) certain clinical conditions that may interfere with CAR values (such as infectious disease, cancer, or hematological disorders); (4) other conditions that may affect bone metabolism (such as estimated glomerular filtration rate (eGFR) < 60 ml/min/1.73m^2^, parathyroid disease, or use of glucocorticoids); (5) OP diagnosis prior to PBC.

In addition, 30 healthy individuals who did not have any of the exclusion criteria were included as controls.

### Data collection

Demographic variables included age, sex, BMI, personal history, and clinical complications. Laboratory investigations included liver biochemical indicators (such as γ-glutamyl transpeptidase and alkaline phosphatase), renal function parameters (eGFR), metabolic parameters (such as total cholesterol and glycated hemoglobin), routine blood tests (such as white blood cells and hemoglobin), and inflammatory indicators (CRP and erythrocyte sedimentation rate).

### Definitions and calculation

Bone mineral density was measured using a dual-energy X-ray absorptiometry (DXA) scanner (Lunar, GE Healthcare, United States). According to the World Health Organization classification for bone mineral density, participants with a T-score ≤ -2.5 at any of the lumbar spine and hip sites were identified as having OP (27).

The Model for End-Stage Liver Disease (MELD) score and Child-Pugh class were calculated based on specific indicators (28, 29).

Fracture Risk Assessment Tool (FRAX) scores (%) were calculated using the Japan FRAX (https://frax.shef.ac.uk/FRAX/tool.aspx?country=2). According to the FRAX score, a high fracture risk was defined as a 10-year probability of major osteoporotic fracture ≥ 20% or a 10-year probability of hip fracture ≥ 3% (30).

### Statistical analysis

#### Propensity score matching analysis

Age, sex, and BMI are recognized risk factors for OP (31); therefore, we adjusted for these three variables using propensity score matching analysis to balance the OP and non-OP groups. Patients with PBC and OP were matched in a 1:3 ratio with those without OP, adopting the nearest neighbor matching algorithm without replacement on the logit of the propensity score with a caliper of width equal to 0.20 standard definitions of the logit of the propensity score. After the propensity score matching analysis, 85 observations (12 in the OP group and 73 in the non-OP group) were trimmed from the lower and upper tails of the propensity score because of the absence of common support. Common support in propensity score matching refers to an overlap in propensity score distribution between the OP and non-OP groups (32). In other words, this is the range of propensity scores including individuals in both the OP and non-OP groups.

### Comparison of variables and construction of models

Continuous variables were expressed as mean ± standard deviation or median (interquartile range) for skewed distributions. Differences between groups were analyzed using Student’s t-test or the Mann–Whitney U test. Categorical variables were presented as percentages (%), and statistical analysis was performed using the chi-square test or Fisher’s exact test. Logistic regression analysis was used to determine the potential risk factors for OP, and the results were presented as odds ratio (OR) and 95% confidence interval (CI). A restricted cubic spline (RCS) was used to explore the relationship between CAR and OP. The predictive value of the variables for OP was assessed using receiver operating characteristic (ROC) curve analysis, and the areas under the curve (AUC) were compared using a nonparametric approach. In practice, there is no standard for classifying AUC values or judging the performance. In general, the accuracy of tests with AUCs between 0.50 and 0.70 is considered low; between 0.70 and 0.90, moderate; and high for AUCs over 0.90 (33). In addition, ROC curve analysis was used to determine the optimal cut-off value for CAR to predict the occurrence of OP. Data analysis was performed using SPSS version 26.0. Figures were generated using GraphPad Prism 9.4.1 and R version 4.1.2. Statistical significance was set at P < 0.05.

## Results

### Baseline characteristics

The baseline characteristics of patients with PBC before and after propensity score matching are shown in **Table 1**. Seventy patients with PBC and OP were matched to 150 patients without OP. In the matched groups, the mean age was approximately 54 years, and the patients were predominantly female.

**Table 1:** Baseline characteristics before and after propensity score matching analysis.

Characteristics	Unadjusted			After propensity score matching		
	OP (n = 82)	Non-OP (n = 223)	P	OP (n = 70)	Non-OP (n = 150)	P
Age, (years)	57.4 ± 11.5	49.9 ± 11.4	<0.001	55.0 ± 10.6	53.0 ± 9.7	0.180
Female, n (%)	78 (95.1)	174 (78.0)	<0.001	66 (94.3)	130 (86.7)	0.091
BMI, (kg/m^2^)	22.5 ± 4.1	23.5 ± 3.4	0.025	23.0 ± 3.3	23.3 ± 3.4	0.491

Values are number (percentage) or mean ± standard deviation. OP, osteoporosis; BMI, body mass index.

**Table 2** shows the demographic and clinical characteristics of the patients after propensity score matching analysis. The proportion of patients with chronic kidney disease was higher in the OP group (28.6% vs. 12.7%, P = 0.004). There was no significant difference in the MELD score and Child-Pugh class between the two groups (P > 0.05). Regarding laboratory indicators, the OP group had lower levels of albumin (37.0 ± 7.3 vs. 40.0 ± 6.0, P = 0.002), eGFR (93.7 ± 17.4 vs. 100.5 ± 15.3, P = 0.003), and hemoglobin (114.0 ± 22.8 vs.122.5 ± 26.4, P = 0.022). However, the inflammatory indicators CRP (12.0 (3.3, 22.0) vs. 3.0 (1.5, 7.3), P < 0.001) and erythrocyte sedimentation rate (20.0 (10.0, 34.0) vs. 10.0 (8.0, 25.5), P < 0.001) were significantly higher in the OP group than in the non-OP group (**Table 3**).

**Table 2:** Demographics and clinical characteristics of patients after propensity score matching analysis.

Characteristics	OP (n = 70)	Non-OP (n = 150)	P
Personal history			
Smoking, n (%)	4 (5.7)	14 (9.3)	0.362
Drinking, n (%)	4 (5.7)	9 (6.0)	0.933
Comorbidities, n (%)			
Hypertension	27 (38.6)	48 (32.0)	0.338
Diabetes mellitus	18 (25.7)	36 (24.0)	0.783
Chronic kidney disease	20 (28.6)	19 (12.7)	0.004
Cerebrovascular disease	5 (7.1)	11 (7.3)	0.960
Cardiovascular disease	4 (5.7)	11 (7.3)	0.657
Disease severity score, n (%)			
MELD score			
<9	49 (70.0)	103 (68.7)	0.842
≥9	21 (30.0)	47 (31.3)	−
Child-Pugh class			
A	47 (65.7)	108 (72.0)	0.572
B	22 (31.4)	37 (24.7)	−
C	2 (2.9)	5 (3.3)	−

Values are number (percentage). OP, osteoporosis; MELD score, Model for End-Stage Liver Disease score. The symbol "-" in the P column indicates that the P value does not need to be reported.

**Table 3:** Laboratory results after propensity score matching analysis.

Characteristics	OP (n = 70)	Non-OP (n = 150)	P
Liver function indicators			
ALT, (U/L)	47.5 (35.8, 76.8)	45.0 (23.8, 105.3)	0.472
AST, (U/L)	51.0 (31.0, 80.3)	47.5 (30.0, 103.3)	0.931
GGT, (U/L)	138.0 (58.5, 269.0)	141.0 (60.5, 277.3)	0.683
ALP, (U/L)	152.3 (94.0, 218.3)	154.0 (97.0, 225.8)	0.898
Direct bilirubin, (μmol/L)	8.3 (4.6, 19.5)	6.5 (4.0, 19.0)	0.196
Albumin, (g/L)	37.0 ± 7.3	40.0 ± 6.0	0.002
Metabolic parameters			
Total cholesterol, (mmoI/L)	4.3 (3.4, 5.3)	4.7 (3.8, 5.8)	0.151
LDL-C, (mmoI/L)	2.4 (1.9, 3.1)	2.5 (2.1, 3.2)	0.173
HbA1c, (%)	5.8 ± 1.0	6.0 ± 1.4	0.409
25-hydroxyvitamin D, (ng/mL)	15.0 (12.0, 20.0)	16.0 (11.0, 21.0)	0.439
Ca^2+^, (mmol/L)	2.2 (2.0, 2.3)	2.3 (2.0, 2.4)	0.694
P, (mmol/L)	1.1 (1.0, 1.2)	1.1 (1.0, 1.3)	0.378
Renal function parameter			
eGFR, (ml/min/1.73m^2^)	93.7 ± 17.4	100.5 ± 15.3	0.003
Blood routine variables			
White blood cell, (10^9/L)	4.8 ± 1.7	5.0 ± 1.6	0.251
Hemoglobin, (g/L)	114.0 ± 22.8	122.5 ± 26.4	0.022
Platelet, (10^9/L)	150.5 (82.8, 190.0)	161.5 (108.0, 242.5)	0.063
Inflammatory indicators			
CRP, (mg/L)	12.0 (3.3, 22.0)	3.0 (1.5, 7.3)	<0.001
ESR, (mm/h)	20.0 (10.0, 34.0)	10.0 (8.0, 25.5)	<0.001

Values are median (interquartile range) or mean ± standard deviation. OP, osteoporosis; ALT, alanine aminotransferase; AST, aspartate aminotransferase; GGT, γ-glutamyl transpeptidase; ALP, alkaline phosphatase; LDL-C, low-density lipoprotein cholesterol; HbA1c, glycated hemoglobin; eGFR, estimated glomerular filtration rate; CRP, C-reactive protein; ESR, erythrocyte sedimentation rate.

### Factors associated with OP in patients with PBC

Among all patients with PBC, the prevalence of OP was 26.9% (82 cases) (**Table 1**). Univariate logistic regression analysis showed that eGFR, hemoglobin, CRP, erythrocyte sedimentation rate, and CAR were statistically significant (P < 0.05). Variables with P < 0.2 were included in the multivariate model for further analysis, except for albumin and CRP, as both were included in the calculation of CAR. We found that patients with PBC with higher eGFR (OR = 0.945, 95%CI = 0.920-0.970, P < 0.001) and hemoglobin levels (OR = 0.984, 95%CI = 0.969-0.999, P = 0.037) had lower odds of OP, suggesting that these metrics may indicate a protective effect. A higher CAR significantly increased the odds of OP (OR = 2.642, 95%CI = 1.537-4.540, P < 0.001). A higher CAR contributed to a 1.64-fold increase in the odds of OP, compared with the slightly reduced odds of OP conferred by eGFR and hemoglobin (**Table 4**).

**Table 4:** Univariable and multivariable logistic regression analysis for predicting OP in patients with PBC.

Variable	Univariate analysis		Multivariate analysis	
	OR (95% CI)	P	OR (95% CI)	P
Demographics and baseline characters				
Hypertension	1.334 (0.739-2.410)	0.339		
Diabetes mellitus	1.096 (0.570-2.108)	0.783		
Cerebrovascular disease	0.972 (0.324-2.913)	0.960		
Cardiovascular disease	0.766 (0.235-2.496)	0.658		
MELD score				
<9	Reference	–		
≥9	0.939 (0.507-1.740)	0.842		
Child-Pugh class				
A	Reference	–		
B-C	1.342 (0.730-2.466)	0.344		
Laboratory findings				
25-hydroxyvitamin D	0.972 (0.928-1.018)	0.225		
GGT	0.999 (0.997-1.000)	0.173	0.999 (0.997-1.001)	0.176
ALP	1.000 (0.999-1.002)	0.347		
Direct bilirubin	0.997 (0.989-1.004)	0.392		
Albumin	0.970 (0.929-1.011)	0.154		
LDL-C	0.795 (0.583-1.084)	0.147	0.953 (0.746-1.218)	0.702
eGFR	0.940 (0.916-0.964)	<0.001	0.945 (0.920-0.970)	<0.001
Hemoglobin	0.988 (0.977-0.998)	0.025	0.984 (0.969-0.999)	0.037
White blood cell	0.835 (0.696-1.002)	0.053	0.977 (0.769-1.241)	0.849
CRP	1.053 (1.019-1.087)	0.002		
ESR	1.015 (1.000-1.030)	0.046	0.988 (0.963-1.013)	0.344
CAR	3.070 (1.798-5.241)	<0.001	2.642 (1.537-4.540)	<0.001

OP, osteoporosis; OR, odds ratio; CI, confidence interval; MELD score, Model for End-Stage Liver Disease score; GGT, γ-glutamyl transpeptidase; ALP, alkaline phosphatase; LDL-C, low-density lipoprotein cholesterol; eGFR, estimated glomerular filtration rate; CRP, C-reactive protein; ESR, erythrocyte sedimentation rate; CAR, C‐reactive protein-to-albumin ratio. The symbol "-" in the P column indicates that the P value does not need to be reported.

### Comparison of bone mineral density between CAR groups

Compared to individuals in the low CAR group, those in the high CAR group had significantly higher bone mineral density at the lumbar spine (0.84 ± 0.13 vs. 0.76 ± 0.14, P < 0.001), right femoral neck (0.85 ± 0.14 vs. 0.71 ± 0.15, P < 0.001), and right total hip (0.84 ± 0.12 vs. 0.75 ± 0.14, P < 0.001) (**Supplementary Table 1**).

### Diagnostic efficacy of CAR for OP in patients with PBC

**Figure 1:**
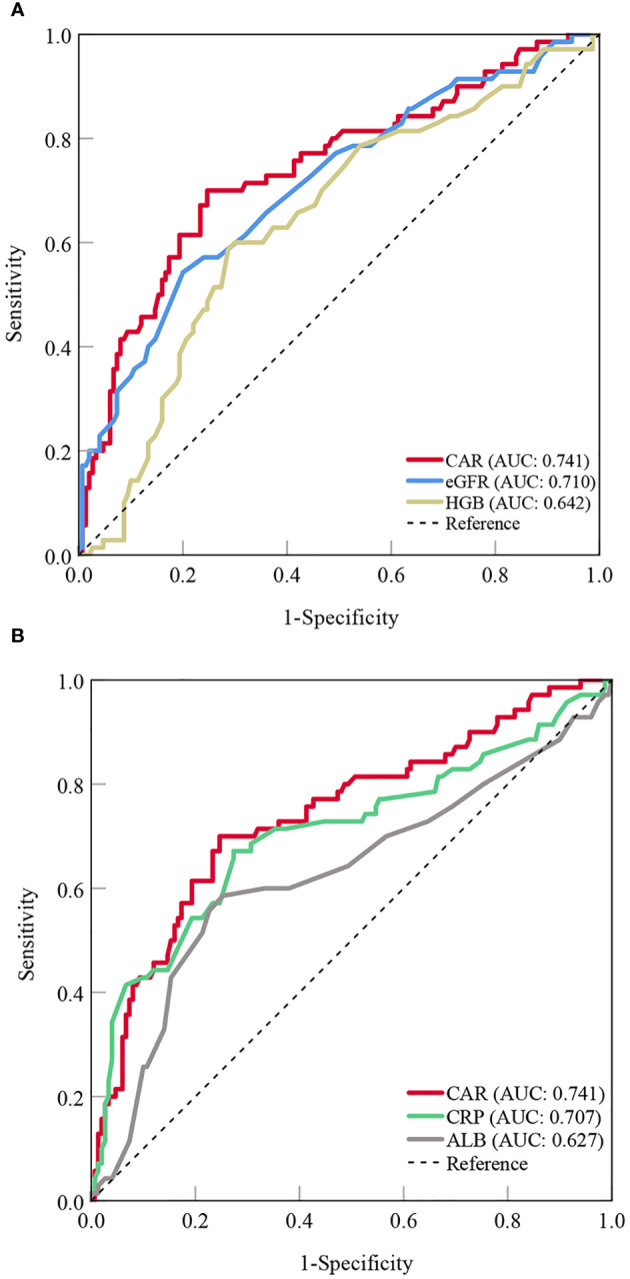
Predictive value of the CAR compared to other indicators for OP in patients with PBC [**(A)** CAR vs. eGFR and HGB; **(B)** CAR vs. CRP and ALB]. CAR, C- reactive protein-to-albumin ratio; OP, osteoporosis; PBC, primary biliary cholangitis; eGFR, estimated glomerular filtration rate; HGB, hemoglobin; CRP, C-reactive protein; ALB, albumin; AUC, areas under the curve.

Based on the results of the multivariate logistic regression analysis, CAR, eGFR, and hemoglobin levels were independent predictors of OP in patients with PBC. The predictive performance of the three indicators for OP in patients with PBC was assessed using AUC. The AUC results were as follows: for CAR, 0.741 (95%CI = 0.678-0.798, P < 0.001); for eGFR, 0.710 (95% CI = 0.645-0.769, P < 0.001); and for hemoglobin, 0.642 (95% CI = 0.575-0.705, P < 0.001). CAR was equally as good as eGFR (ΔAUC: 0.031, P = 0.555), but significantly better than hemoglobin (ΔAUC: 0.099, P = 0.043) in predicting OP in patients with PBC. We also analyzed the predictive values of CRP and albumin levels for OP. The results showed that the predictive ability of CAR was superior to both CRP (ΔAUC: 0.034, P = 0.017) and albumin (ΔAUC: 0.114, P = 0.002) (**Figures 1A, B**, **Table 5**).

**Table 5:** Predictive value of CAR versus other indicators for OP.

	Single AUC analysis			Difference between AUC			
	AUC	95%CI	P	Δ AUC	95%CI	Z statistic	P
CAR	0.741	0.678-0.798	<0.001	Reference	…	…	…
eGFR	0.710	0.645-0.769	<0.001	0.031	(-0.072)-0.133	0.590	0.555
Hemoglobin	0.642	0.575-0.705	<0.001	0.099	0.003-0.195	2.021	0.043
CRP	0.707	0.642-0.766	<0.001	0.034	0.006-0.0616	2.385	0.017
Albumin	0.627	0.559-0.691	0.004	0.114	0.042-0.187	3.080	0.002

CAR, C‐reactive protein-to-albumin ratio; OP, osteoporosis; AUC, areas under the curve; CI, confidence interval; eGFR, estimated glomerular filtration rate; CRP, C-reactive protein.

**Figure 2:**
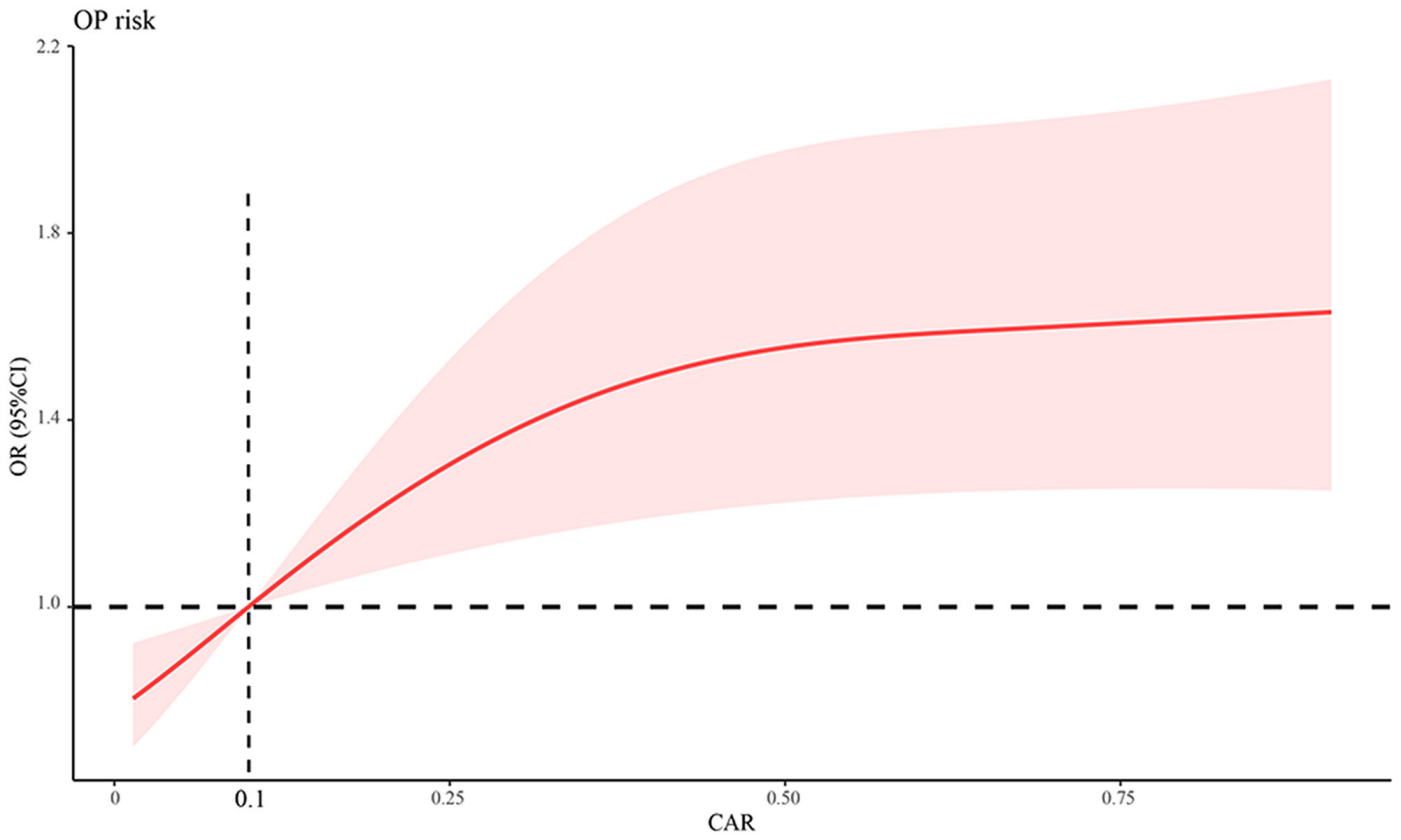
Association between CAR and OP odds using a RCS regression model. CAR, C‐reactive protein-to-albumin ratio; OP, osteoporosis; RCS, restricted cubic spline; OR, odds ratio; CI, confidence interval.

Furthermore, we used a three-point RCS to examine the association between the CAR and the odds of OP in patients with PBC. A linear relationship was observed between CAR and OP. In other words, a higher CAR was associated with higher odds of developing OP in patients with PBC. We found a significant increase in the odds of OP when CAR was > 0.1, as shown in **Figure 2**.

### CAR is associated with the risk of OP and fracture

**Figure 3:**
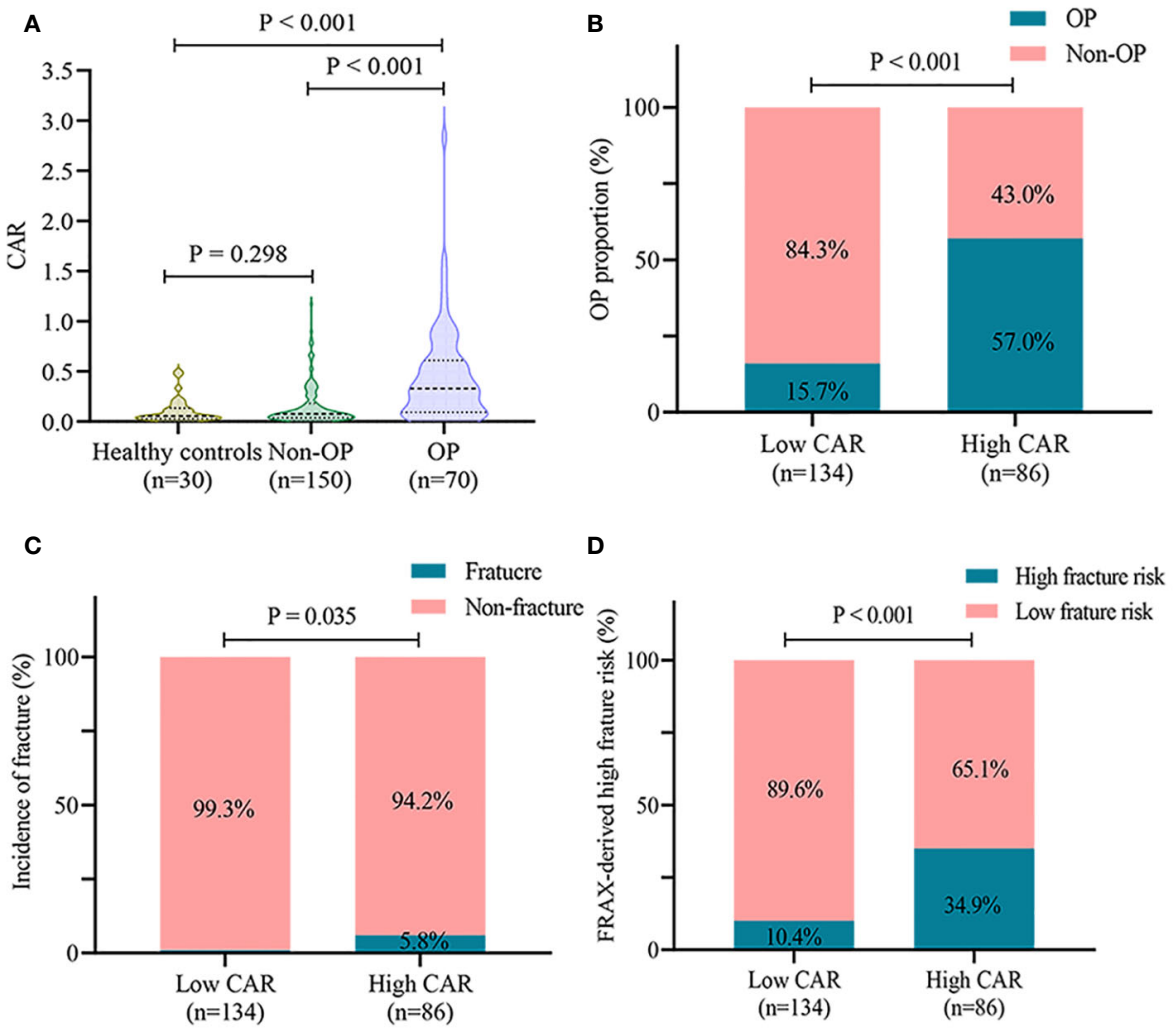
CAR is associated with OP in patients with PBC. **(A)** comparison of CAR levels in healthy controls, non-OP, and OP groups; **(B)** incidence of OP in different CAR groups; **(C)** incidence of fractures in different CAR groups; **(D)** high 10-year fracture risk as derived from FRAX in different CAR groups. CAR, C-reactive protein-to-albumin ratio; OP, osteoporosis; PBC, primary biliary cholangitis; FRAX, Fracture Risk Assessment Tool.

As shown in **Figure 3A**, CAR levels were higher in the OP group than in the non-OP group (0.33 (0.09, 0.61) vs. 0.08 (0.04, 0.18), P < 0.001) and healthy controls (0.33 (0.09, 0.61) vs. 0.05 (0.03, 0.13), P < 0.001). There was no significant difference in CAR values between patients without OP and healthy controls (0.08 (0.04, 0.18) vs. 0.05 (0.03, 0.13), P = 0.298). The cut-off value of CAR for OP was 0.18, with a sensitivity of 0.70 and specificity of 0.75. The patients were divided into two groups according to the cutoff values: low CAR (134 cases) and high CAR (86 cases). The high CAR group had a higher incidence of OP than the low CAR group (57.0% vs. 15.7%, P < 0.001) (**Figure 3B**). Furthermore, the high CAR group had a higher prevalence of fragility fractures (5.8% vs. 0.7%, P = 0.035) and a higher 10-year fracture risk as derived from FRAX (34.9% vs. 10.4%, P < 0.001) (**Figures 3C, D**).

### Prevalence of OP according to different disease stages

Because the risk of OP may be related to the severity of liver disease (34), we compared the rates of OP at different stages of disease progression. However, our results showed that the prevalence of OP was not significantly higher in patients with an advanced MELD score (32.2% vs. 30.9%, P = 0.842) or Child-Pugh class (29.9% vs. 37.3% vs. 28.6%, P = 0.527) (**Supplementary Figures 1A, B**).

## Discussion

The main results of this study are as follows: (1) the prevalence of OP was 26.9% (82 cases) in patients with PBC; (2) CAR was an independent predictor of OP in patients with PBC (OR = 2.642, 95%CI = 1.537-4.540), and the odds of OP increased significantly when CAR was > 0.1; (3) CAR (AUC: 0.741) was equally as good as eGFR (ΔAUC: 0.031, P = 0.555), but significantly better than hemoglobin (ΔAUC: 0.099, P = 0.043), CRP (ΔAUC: 0.034, P = 0.017) and albumin (ΔAUC: 0.114, P = 0.002) in predicting OP in patients with PBC; (4) high CAR levels were associated with an increased prevalence of OP and fragility fractures, and a high 10-year fracture risk as derived from FRAX.

Osteopenic bone disease, a condition of reduced bone mineral density and strength, is a common extrahepatic complication of PBC (35, 36). Most patients with PBC have osteopenia, and 20–44% have OP (1, 37). In our study, the incidence of OP in patients with PBC was 26.9%, which is comparable to previous findings. OP and associated fragility fractures can lead to impaired physical function and poor prognosis (38). Therefore, it is important to assess the risk of OP and initiate the appropriate treatment as early as possible to prevent disease progression.

Inflammation is known to impair bone metabolism, and pro-inflammatory markers and cytokines are potent inducers of osteoclastogenesis (39–41). CRP, a commonly used clinical marker of systemic inflammation, is linked to the development of metabolic bone diseases (42). In a systematic review and meta-analysis, Mun et al. (12) showed that high CRP levels are associated with a significantly increased risk of osteoporotic fractures. However, some studies have questioned the utility of CRP levels in predicting bone health. Researchers have found that serum CRP levels do not correlate with plasma vitamin D concentrations, and cannot be used as an indicator of bone loss (43, 44). In addition, a recent study in a large sexually and racially diverse sample also suggested that although CRP correlated with and predicted bone mineral density, the small magnitude had no biological significance (45). Similar to CRP, the role of albumin in bone metabolism remains controversial. Saito et al. (46) found that decreased serum albumin levels positively correlated with the degree of decrease in peak bone mineral density in healthy individuals. Similarly, a large study of 21121 patients reported an independent association between OP and lower albumin levels and a longer duration of hypoalbuminemia (14). In contrast, Lunde et al. (47) showed a weak correlation between circulating albumin levels and bone mineral density, which disappeared after adjusting for age. These inconsistencies suggest that relying on a single indicator, CRP or albumin, may not accurately predict the risk of OP in different study contexts (48).

The CAR is the ratio of CRP to albumin and was first proposed by Fairclough et al. (49) to predict outcomes in acute admissions. As a composite indicator of inflammation and nutrition, the CAR has been linked to several pathological conditions, including severe infections, ulcerative colitis, and clinical activity and deterioration of tumors (50–52). In this study, we found that the CAR was significantly higher in patients with PBC and OP and could be used as an independent predictor of OP. The predictive ability of CAR for OP was superior to that of single indicator, CRP or albumin. Consistent with our observations, Yang et al. (23) found that the predictive performance of CAR for cardiovascular disease was better than that of CRP or albumin alone. In addition, our results showed that hemoglobin and eGFR were independently associated with odds of developing OP in patients with PBC. These two serum indicators have been reported to play a protective role against OP (53, 54). Xiu et al. (55) found a positive association between hemoglobin level and bone mineral density in older Chinese individuals with type 2 diabetes mellitus. Similarly, a study in Japan showed that postmenopausal women with mild renal dysfunction had an increased risk of OP and vertebral fractures (56). Further ROC analysis showed that the predictive ability of CAR for OP was similar to that of eGFR but better than that of hemoglobin. Thus, these results indicate the feasibility and accuracy of CAR in predicting OP.

Previous studies have shown that patients with severe liver disease have an increased incidence of low bone mineral density and osteoporotic fractures (34). Advanced fibrosis and cirrhosis have been linked to poor bone mineral density in non-alcoholic fatty liver disease (57). We investigated the relationship between liver disease severity and OP in patients with PBC. The results showed that the prevalence of OP in patients with a high Child-Pugh class or MELD score was similar to that in patients with a low score. Similar to our results, Chinnaratha et al. (58) found no direct association between liver disease severity (measured by the MELD score and Child-Pugh class) and hepatic osteodystrophy in patients with newly diagnosed cirrhosis. The inconsistency in these findings may be related to different study contexts and populations. Another possible explanation is that we excluded patients with end-stage liver disease from this study, and the prevalence of OP in patients with severe PBC may have been underestimated.

Patients with PBC have an increased risk of fractures compared with the general population, with a hazard ratio of 1.9 (36, 59). Six patients in our study population had fractures (vertebral, n = 4; rib, n = 2), five of which occurred in the OP group. We also compared the risk of fractures between the different CAR groups. High CAR values are not only associated with an increased fracture incidence, but also with a high 10-year fracture risk as derived from FRAX. Long-term fracture risk in patients with liver disease may be underestimated using bone mineral density alone. Our results suggest that the CAR may complement bone mineral density in the assessment of long-term fracture risk. Guidelines recommend screening for bone diseases in patients with chronic liver disease (60). However, clinical adherence to these recommendations is unclear, and whether screening and early intervention are cost-effective in preventing OP and fragility fractures in patients with PBC is unknown. To the best of our knowledge, this is the first study to show that high CAR levels are positively associated with OP in patients with PBC. Targeted screening of the high-risk population identified in our study (individuals with a higher CAR) may be a potential strategy to improve cost-effectiveness and patient prognosis.

Based on only two measured parameters, the CAR has the advantages of broad applicability and low cost. In addition, it is easy to use and does not require specialized knowledge for interpretation (52). We found that a higher CAR contributed to a 1.64-fold increase in the odds of OP, and the predictive value of the CAR for OP was superior to that of the other examined indicators. However, it must be acknowledged that the AUC of 0.741 still limits its statistical validity. The suboptimal predictive power of CAR may be related to the small sample size (220 patients) of the study. The small sample size may have reduced the statistical power of the CAR and the ability to detect the true odds of OP. In addition, false negative results may have been obtained in the present study. On the one hand, the diagnosis of OP based on an absolute bone mineral density T-score ≤ -2.5 may fail to identify individuals with potential OP (T-score close to -2.5); on the other hand, we did not assess the diagnostic value of CAR in patients with osteopenia (-2.5 < T-score < -1). However, CAR was independently correlated with OP after adjusting for multivariate logistic regression. In other words, the observed association between the CAR and OP is not an epiphenomenon, and the CAR could serve as an independent predictor of OP in patients with PBC. Therefore, CAR may be considered an alternative for the initial screening of OP in patients with PBC, particularly in resource-limited settings. This study may help clinicians identify patients with PBC at high risk of OP early and promptly intervene to prevent disease progression and improve prognosis. However, further studies with larger sample sizes are needed to better understand the pathophysiological relationship between CAR and OP, and to determine the applicability of CAR for OP screening in clinical practice.

Some limitations should be considered. First, the number of included studies investigating the association between the CAR and OP was small, which makes our findings regarding the CAR less conclusive and comprehensive. Therefore, future studies need to increase the sample size to further investigate the relationship between CAR and metabolic bone diseases and validate its diagnostic value with long-term follow-up. Second, we did not measure serum bone-turnover markers such as osteocalcin, PINP and β-CTX. However, the high testing costs limit the widespread use of these indicators in clinical practice. In contrast, the diagnosis of osteopenic bone disease is mainly based on bone mineral density measured using DXA. This was a retrospective real-world study, and because we did not collect and store blood samples from patients during their early visits, we were unable to additionally test for these indicators. In addition, patients with end-stage liver disease were not included in our study; therefore, the results need to be validated in all PBC populations. Finally, medications that may affect bone mineral density were not analyzed. These drugs were not evaluated in the present study because of their limited use in patients with PBC.

## Conclusion

High CAR levels are associated with greater odds of developing OP, and CAR can be used as a biomarker to predict OP in patients with PBC. The incidence of OP increased significantly when the CAR was > 0.1. Therefore, clinicians should focus on patients with PBC with high CRP and low albumin levels to prevent the development of OP and fractures.

## Data availability statement

The original contributions presented in the study are included in the article/**Supplementary Material**. Further inquiries can be directed to the corresponding author.

## Ethics statement

The studies involving humans were approved by Institutional Review Board of Beijing Ditan Hospital. The studies were conducted in accordance with the local legislation and institutional requirements. Written informed consent for participation was not required from the participants or the participants’ legal guardians/next of kin in accordance with the national legislation and institutional requirements.

## Author contributions

YL: Conceptualization, Data curation, Methodology, Writing – original draft, Writing – review & editing. BL: Formal analysis, Methodology, Writing – review & editing. XL: Resources, Supervision, Validation, Writing – review & editing.

## Supplementary material

Supplementary Figure 1 Incidence of OP according to the MELD score **(A)** and Child-Pugh class **(B)**. OP, osteoporosis; MELD score, Model for End-Stage Liver Disease score.

## Glossary

**Table:**

CAR	C-reactive protein-to-albumin ratio
OP	osteoporosis
PBC	primary biliary cholangitis
ROC	receiver operating characteristic
RCS	restricted cubic spline
eGFR	estimated glomerular filtration rate
DXA	dual-energy X-ray absorptiometry
MELD score	Model for End-Stage Liver Disease score
FRAX	Fracture Risk Assessment Tool
OR	odds ratio
CI	confidence interval
AUC	areas under the curve
ALT	alanine aminotransferase
AST	aspartate aminotransferase
GGT	γ-glutamyl transpeptidase
ALP	alkaline phosphatase
LDL-C	low-density lipoprotein cholesterol
HbA1c	glycated hemoglobin
eGFR	estimated glomerular filtration rate
ESR	erythrocyte sedimentation rate
HGB	hemoglobin
CRP	C-reactive protein
ALB	albumin
BMI	body mass index.
