# Supplementary figures and images for: High C-reactive protein-to-albumin ratio levels are associated with osteoporosis in patients with primary biliary cholangitis
Source: Front Endocrinol (Lausanne). 2024 May 30;15:1415488. doi: 10.3389/fendo.2024.1415488 (PMC11169652; doi:10.3389/fendo.2024.1415488)

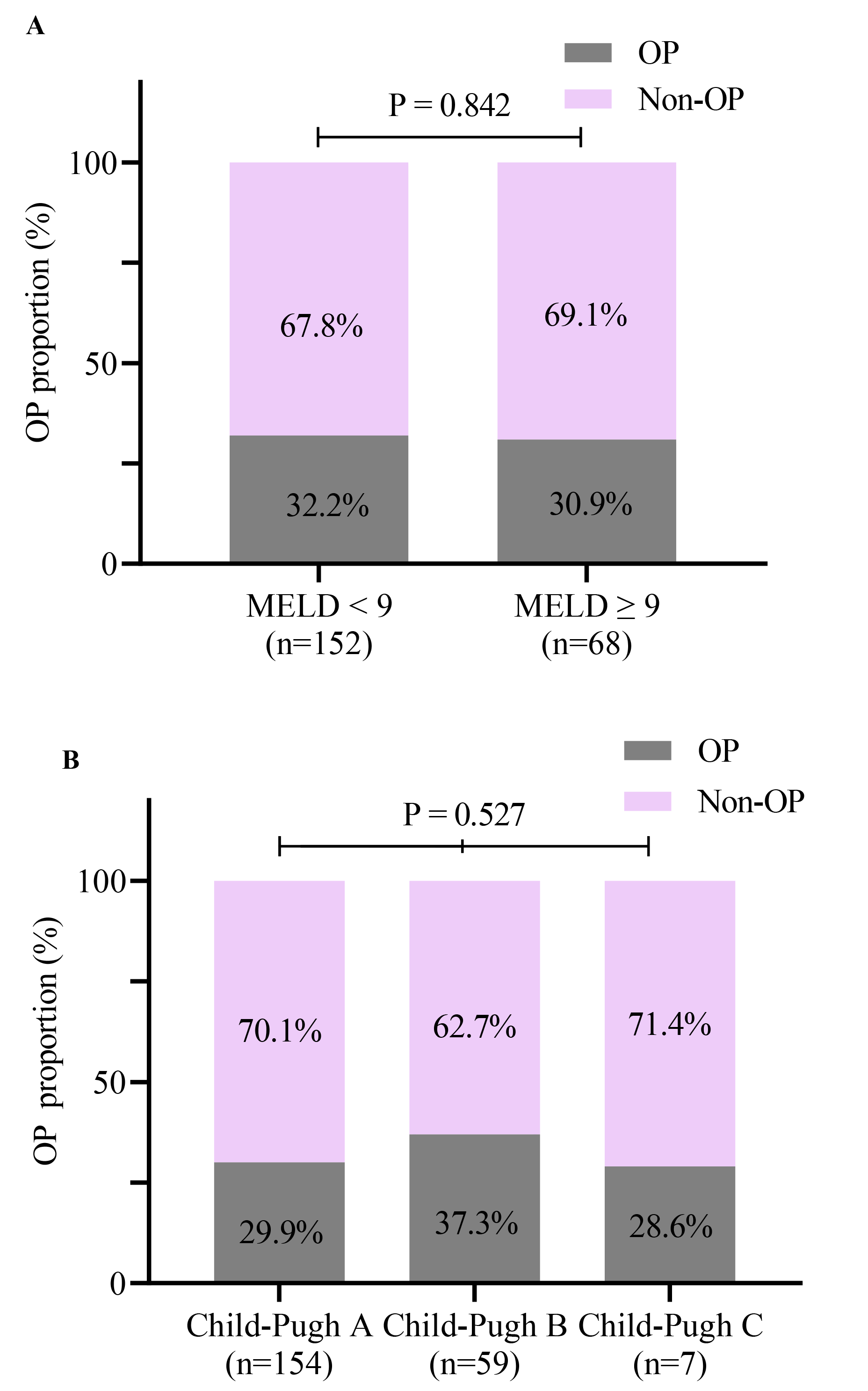

Supplement: Supplementary Figure 1 — Incidence of OP according to the MELD score (A) and Child-Pugh class (B). OP, osteoporosis; MELD score, Model for End-Stage Liver Disease score. [file Image_1.tif]
