# Supplementary material for: High C-reactive protein-to-albumin ratio levels are associated with osteoporosis in patients with primary biliary cholangitis
Source: Front Endocrinol (Lausanne). 2024 May 30;15:1415488. doi: 10.3389/fendo.2024.1415488 (PMC11169652; doi:10.3389/fendo.2024.1415488)
Supplement: Supplementary file 2 [file Table_1.docx]

**Supplementary Table 1 Comparison of bone mineral density between CAR groups**

| Characteristics | Low CAR (n = 134 ) | High CAR (n = 86) | P |
| --- | --- | --- | --- |
| Dual-energy X-ray absorptiometry |  |  |  |
| Bone mineral density, (g/cm^2^) |  |  |  |
| Lumbar spine | 0.84 ± 0.13 | 0.76 ± 0.14 | <0.001 |
| Right femoral neck | 0.85 ± 0.14 | 0.71 ± 0.15 | <0.001 |
| Right total hip | 0.84 ± 0.12 | 0.75 ± 0.14 | <0.001 |
| T-score |  |  |  |
| Lumbar spine | -0.70 (-1.75, 0.40) | -2.60 (-3.30, -0.68) | <0.001 |
| Right femoral neck | -0.50 (-1.80, 0.30) | -2.82 (-3.60, -0.50) | <0.001 |
| Right total hip | -0.70 (-1.83, 0.30) | -2.00 (-2.93, -0.68) | <0.001 |

Abbreviations: Values are median (interquartile range) or mean ± standard deviation. CAR, C‐reactive protein-to-albumin ratio.
